# Supplementary material for: Dynamic Phosphorylation of miRNA Biogenesis Factor HYL1 by MPK3 Involving Nuclear–Cytoplasmic Shuttling and Protein Stability in Arabidopsis
Source: Int J Mol Sci. 2022 Mar 30;23(7):3787. doi: 10.3390/ijms23073787 (PMC8998556; doi:10.3390/ijms23073787)
Supplement: Supplementary file 1 [file ijms-23-03787-s001.zip › ijms-1606189-supplementary.pdf]

Supplementary Information

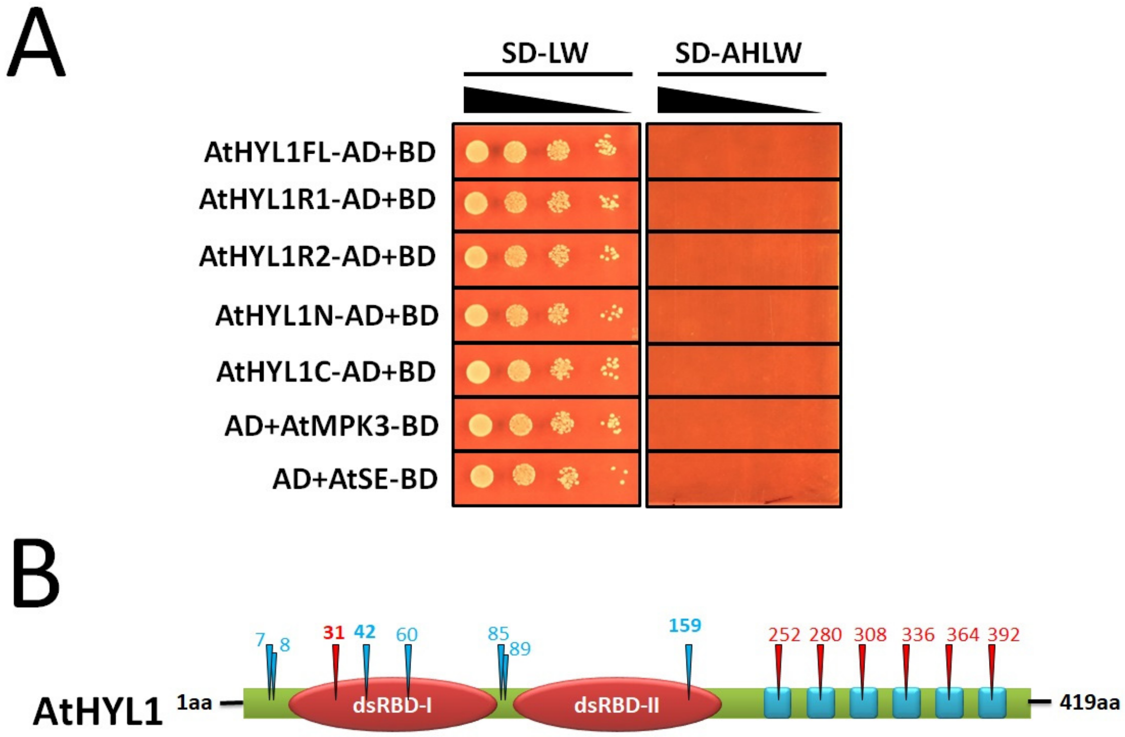

Figure S1. Controls of Y2H assay (Related to Figure 1.) (**A**) Protein-protein interaction using empty vectors with AtHYL1 and its variants along with AtMPK3 and AtSE in SD-LW and SD-AHLW plates. (**B**) Distribution of putative phosphorylation sites in AtHYL1 as detected by NetPhos. Blue and red arrow head represents the potential serine and threonine phosphorylation residues.

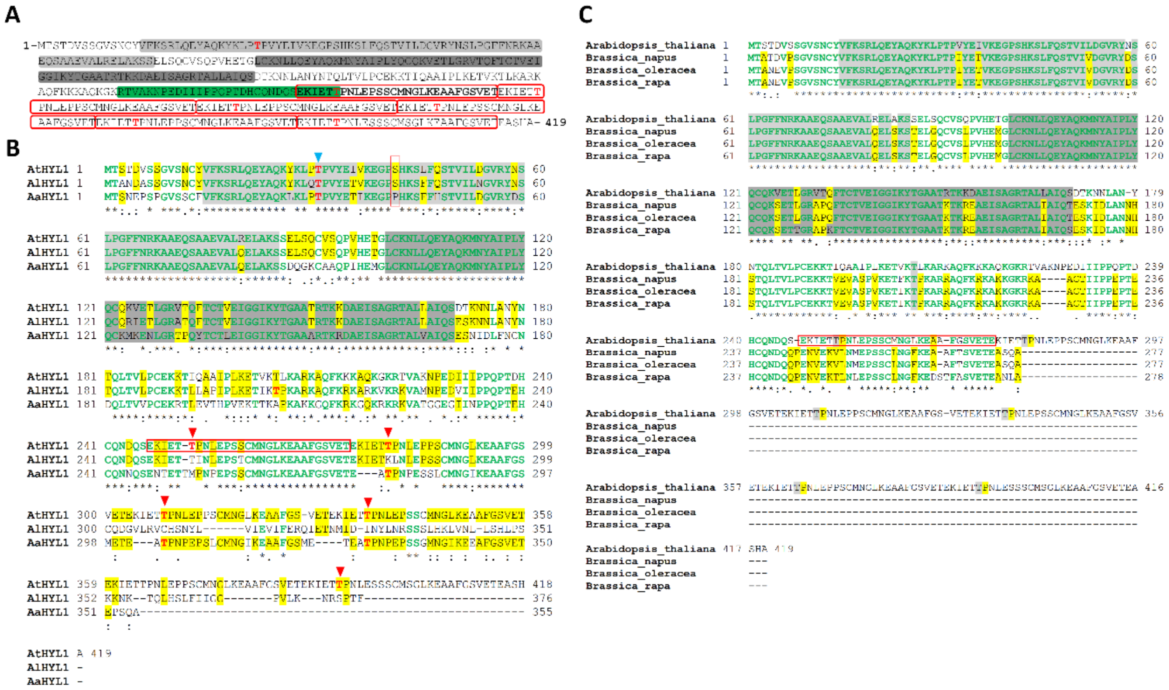

Figure S2. (**A**) Protein sequence of AtHYL1. Two dsRBDs are highlighted by light and dark grey colour. Bi-partite NLS is highlighted in green colour. The 28 amino acid repeat at C-terminal is in red box. The canonical MAP kinase phosphorylation site, i.e. threonine is in red colour. (**B**) Multiple protein sequence alignment of AtHYL1 and its close members *Arabidopsis lyrata* (Al) and *Arabidopsis alpine* (Aa). Blue arrow head indicates the evolutionarily conserved canonical MAP kinase phosphorylation site and red arrow heads represent other putative MAP kinase target sites. A non-canonical MAP kinase site, serine-42 is indicated in red box. (**C**) Multiple protein sequence alignment of AtHYL1 and its close members of Brassica.

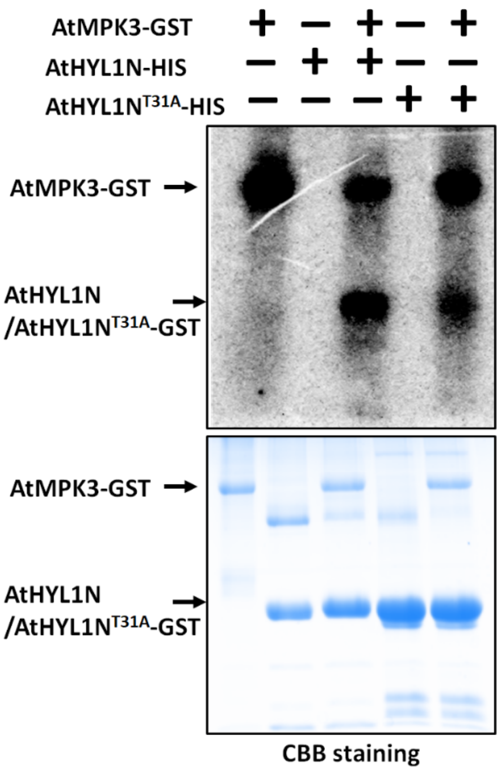

Figure S3. *In-vitro* phosphorylation assay using a bacterially purified AtHYL1N wild type and AtHYL1N^{T31A} by AtMPK3. The canonical evolutionarily conserved threionine-31 was mutated to alanine, a non-phosphorytable amino acid.

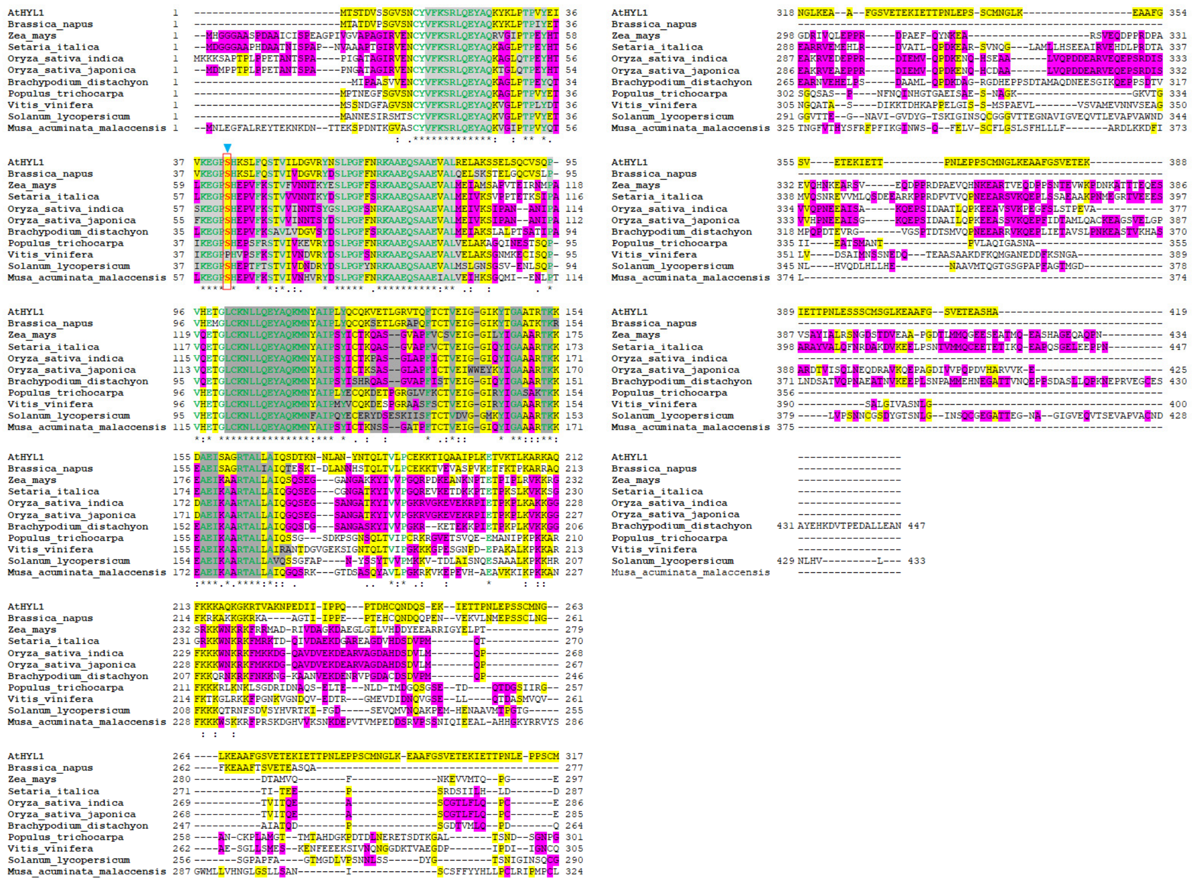

Figure S4. Multiple protein sequence alignment showing the evolutionarily conservation of serine-42 of AtHYL1 in other plant species indicated by blue arrow head within red box.

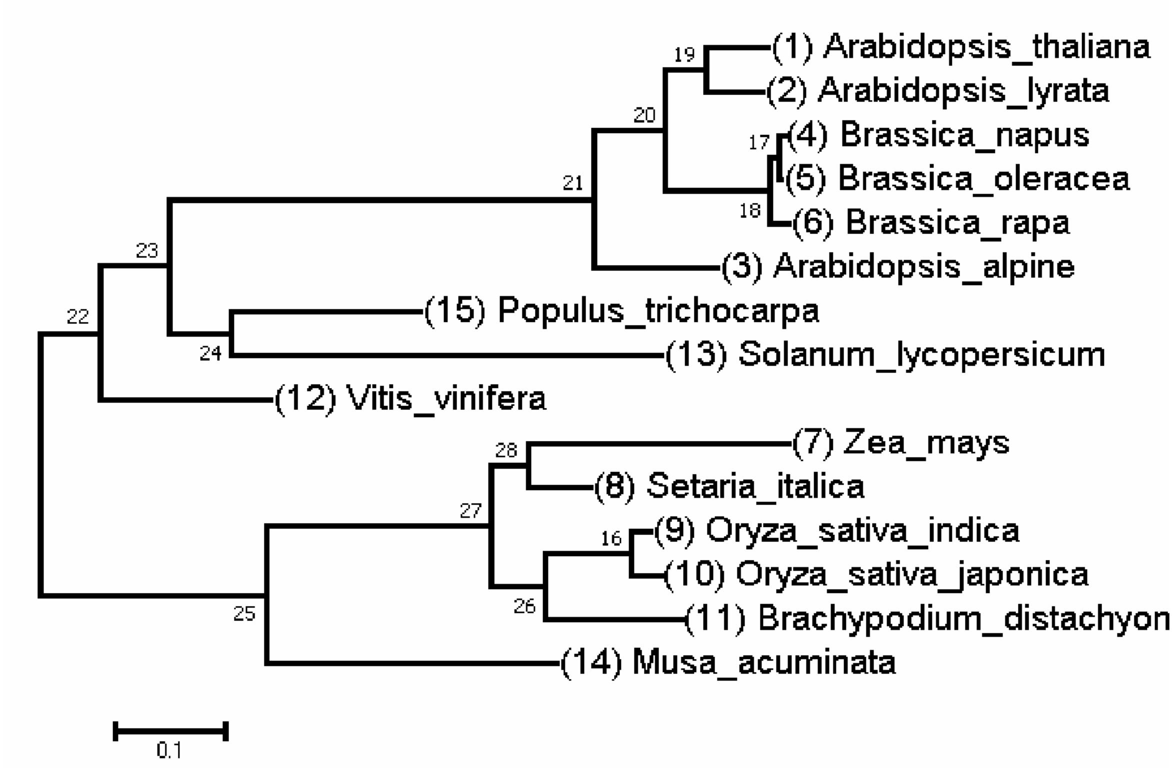

Figure S5. Phylogenetic tree showing the relation of AtHYL1 with its homologue proteins in other plants.

A

```
1- MTSTDVSSGVSNCYVFKSRLQEYAQKYKLPTPVYEIVKEGPSHKSLFQSTVILDGV
   RYNSLPGFFNRKAAEQSAAEVALRELAKSSELSQCVSQPVHETGLCKNLLQEYAQK
   MNYAIPLYQCQKVETLGRVTQFTCTVEIGGIKYTGAATRTKKDAEISAGRTALLAI
   QSDTKNNLANYNTQLTVLPCEKKTIQAAIPLKETVKTLKARKAQFKKKAQKGKRTV
   AKNPEDIIIPPQPTDHCQNDQSEKIETTPNLEPSSCMNGLKEAAFGSVETEKIETT
   PNLEPPSCMNGLKEAAFGSVETEKIETTPNLEPPSCMNGLKEAAFGSVETEKIETT
   PNLEPSSCMNGLKEAAFGSVETEKIETTPNLEPPSCMNGLKEAAFGSVETEKIETT
   PNLESSSCMSGLKEAAFGSVETEASHA-419
```

B

Cleavage site	N-terminal Native (kDa)	C-terminal Native (kDa)
R-222	24.6	20.9
K-226	25	20.5
K-248	27.5	18.4
K-265	29.3	16.2
K-276	30.4	15
K-293	32.3	13.2
K-304	33.4	12.1
K-321	35.2	10.2
K-332	36.4	9.1
K-349	38	7.3
K-360	39.4	6.1
K-377	41.2	4.3
K-388	42.3	3.1
K-405	44.1	1.4

Figure S6. (**A**) AtHYL1 protein sequence showing the presence of putative protease site for trypsin like protease. The multiple lysine (K) residues are highlighted by green and multiple putative MAP kinase sites threonine by red colour. A single arginine (R) residue is present within the NLS indicated with red colour. (**B**) The putative molecular weight of cleaved peptide at indicated target sites are presented in the tabular form.

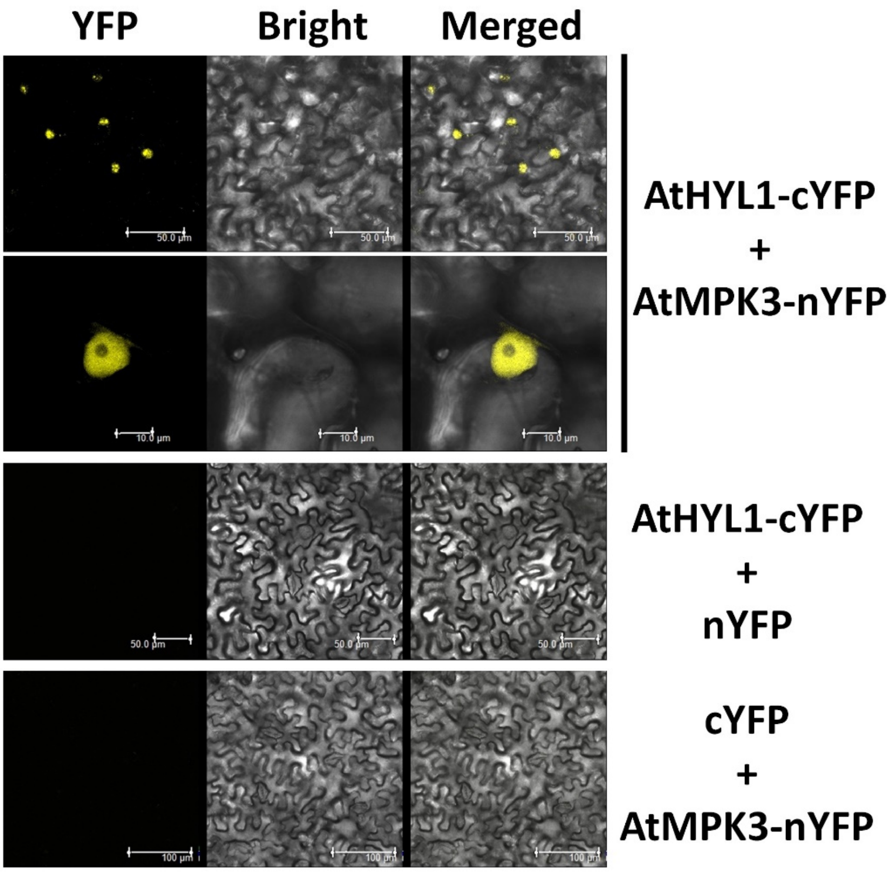

Figure S7. BiFC assay showing the interaction between AtHYL1-cYFP and AtMPK3-nYFP in *N. benthamiana* leaves under confocal microscope.

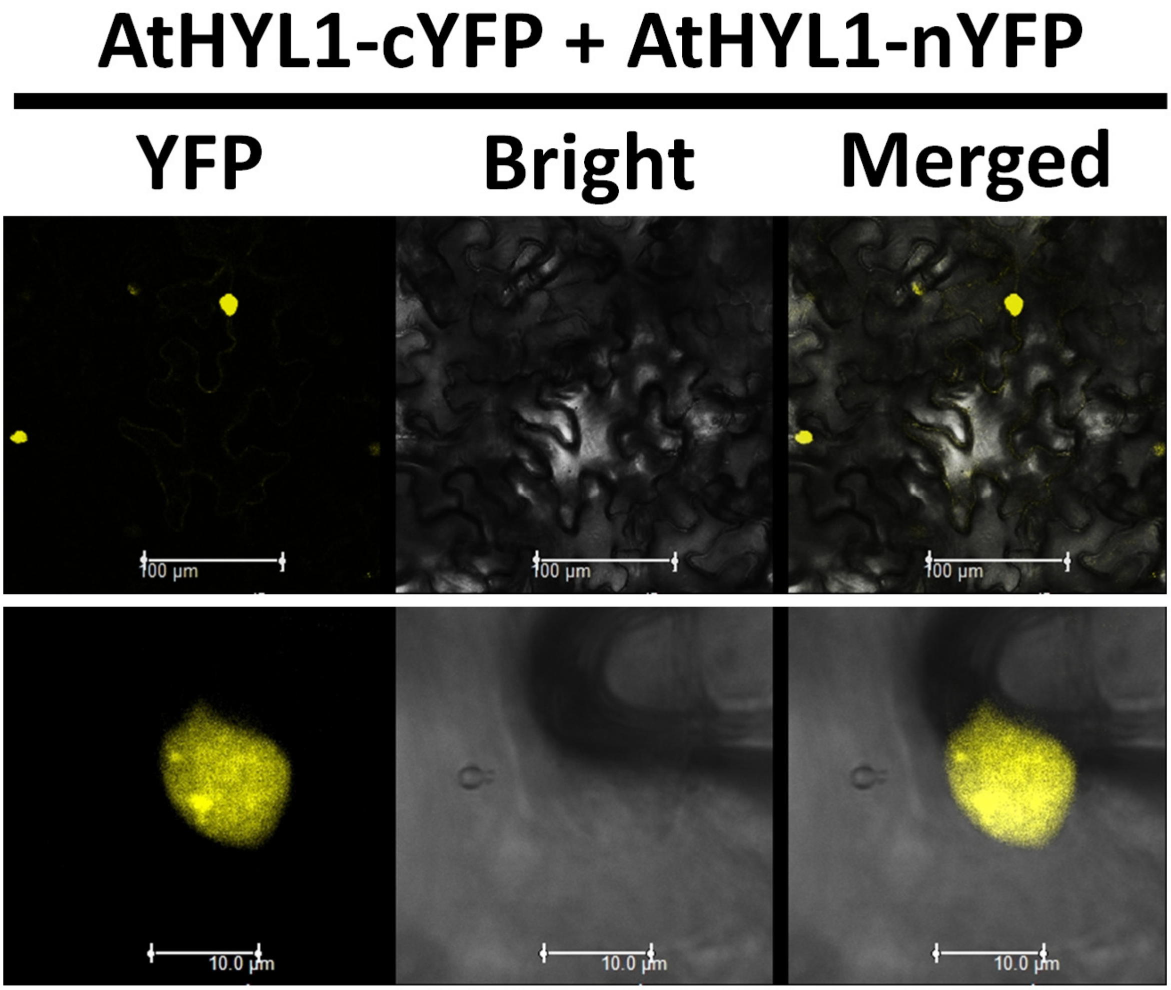

Figure S8. BiFC assay showing the interaction between AtHYL1-cYFP and AtHYL1-nYFP in *N. benthamiana* leaves under confocal microscope.

Table S1: Putative phosphorylation sites on AtHYL1. Phosphorylation -sites of AtHYL1 protein were predicted by the program of NetPhos 2.0 server (http://www.cbs.dtu.dk/services/NetPhos-2.0/). All possible threonine and serine sites of phosphorylation are given. Yellow highlighted are the putative MAP kinase phosphorylation sites.

No	Position	Group	score
**Threonine**			
1	2	---M**T**STDV	0.631
2	31	YKLP**T**PVYE	0.313
3	152	AATR**T**KKDA	0.936
4	205	ETVK**T**LKAR	0.818
5	252	KIET**T**PNLE	0.845
6	274	GSVE**T**EKIE	0.851
7	280	KIET**T**PNLE	0.845
8	302	GSVE**T**EKIE	0.851
9	308	KIET**T**PNLE	0.845
10	330	GSVE**T**EKIE	0.851
11	336	KIET**T**PNLE	0.845
12	358	GSVE**T**EKIE	0.851
13	364	KIET**T**PNLE	0.845
14	386	GSVE**T**EKIE	0.851
15	392	KIET**T**PNLE	0.845
		**Serine**	
1	7	STDV**S**SGVS	0.629
2	8	TDVS**S**GVSN	0.808
3	42	KEGP**S**HKSL	0.987
4	60	VRYN**S**LPGF	0.729
5	85	ELAK**S**SELS	0.973
6	89	SSEL**S**QCVS	0.815
7	159	DAEI**S**AGRT	0.897

Table S2: List of primers used in the present study

SN	Gene	Sequence	Use
1	AtMPK3	For: ATCCGGAATTCATGAACACCGGCGGTGG<br>Rev: ATCGCGGATCCCCTAACCGTATGTTGGATTGAGTGC	Y2H
2	AtHYL1/DRB1	For: CGGGAATTCATGACCTCCACTGATGTTTC<br>Rev: CCGCTCGAGTGCGTGGCTTGCTTC	Protein Expression for in-vitro phosphorylation assay
	AtHYL1-N	For: CGCGGATCCGCATGACCTCCACTGATGTTTC<br>Rev: ATCCGCTCGAGTGACTGGATCGCTAAAAGAG	
	AtHYL1-C	For: CGCGGATCCGCGACACTAAAAACAACCTTG<br>Rev: GTCCGGAATTCTTATGCGTGGCTTGCTTCTGTCTCC	
	AtHYL1-N	For: GAATTCATGACCTCCACTGATGTTTC<br>Rev: CTCGAGAGGAAGTACAGTAAGCTGAGTG	Protein expression
	AtHYL1-RDM-I	For: CTGCATATGACCTCCACTGATGTTTC<br>CGGGAATTCATGACCTCCACTGATGTTTC<br>Rev: CGGGAATTCTTGTGAAACACATTGGCTTAG	Y2H
	AtHYL1-RDM-II	For: CTGCATATGTTCACGAAACGGATTATGC<br>Rev: CGGGAATTCAGGAAGTACAGTAAGCTGAGTG	
	AtHYL1-C	For:CTGCATATG TGTGAGAAGAAGACAATACAG<br>Rev: CGGGAATTC GACACTGTTATGCGTGGCTTG	
3	AtSE	For: ATCCGGAATTCATGGCCGATGTTAATCTTCCTCC<br>Rev: ATCGCGGATCCCCTACAAGCTCCTGTAATCAATAACGG	
4	OsMPK3	For: GGATCCATGGGGATGGACGGGGCGCCGGTG<br>Rev:CGGAATTCCGCTAGTACCGGATGTTTGGGTTCATCTCGAT	Protein expression
5	OsDRB1-1	For: ATCGCGGATCCATGAAGAAAAAAAGTGCTCCC<br>Rev: GTCCGGAATTCTCAGGCTACCTCAGGTGTTG	Protein expression
	OsDRB1-1-N	For: CGCGGATCCGCATGAAGAAAAAAAGTGCTC<br>Rev: ATCCGCTCGAGACCTTGGATTGCCAGAAGAG	
	OsDRB1-1-C	For: CGCGGATCCGCCAATCAGAGGGTTCTGCAAATG<br>Rev: GTCCGGAATTCTCAGGCTACCTCAGGTGTTG	
6	OsDRB1-2	For: CGCGGATCCGCATGGACATGCCGCCCAC<br>Rev: CCGCTCGAGTTCTTCGCTCATATTAGT	
	OsDRB1-2-N	For: CGCGGATCCGCATGGACATGCCGCCCAC<br>Rev: ATCCGCTCGAGACCTTGGATTGCCAGAAGAG	
	OsDRB1-2-C	For: CGCGGATCCGCCAATCAGAGGGTTCTGCAAATG<br>Rev: CCGCTCGAGTTCTTCGCTCATATTAGT	
7	OsDRB1-4	For: CGCGGATCCGCATGGCGGCCGCCACCGCC<br>Rev: CCGCTCGAGCTGTGCAACTCTTTCTTC	
	OsDRB1-4-N	For: CGCGGATCCGCATGGCGGCCGCCACCGCC<br>Rev: ATCCGCTCGAGAGCTAAAAGTGACTTGACCG	
	OsDRB1-4-C	For: CGCGGATCCGCACAAATTACACTTCCATG<br>Rev: CCGCTCGAGCTGTGCAACTCTTTCTTC	
8	AtHYL1	For: CACCATGACCTCCACTGATGTTTC	Gateway

		Rev: TGCGTGGCTTGCTTCTG	Cloning in pENTR
